# Effects of family-based dignity intervention and expressive writing on anticipatory grief of family caregivers of patients with cancer: a study protocol for a four-arm randomized controlled trial and a qualitative process evaluation

**DOI:** 10.1186/s13063-021-05718-3

**Published:** 2021-10-28

**Authors:** Naima Seyedfatemi, Tahereh Najafi Ghezeljeh, Jafar Bolhari, Masoud Rezaei

**Affiliations:** 1 Nursing Care Research Center, Iran University of Medical Sciences, Tehran, Iran; 2 Nursing Care Research Center, School of Nursing and Midwifery, Iran University of Medical Sciences, Tehran, Iran; 3 Spiritual Health Research Center, School of Behavioral Sciences and Mental Health, Iran University of Medical Sciences, Tehran, Iran

**Keywords:** Anticipatory grief, Caregivers, Cancer, Dignity, Expressive writing

## Abstract

**Background:**

Family caregivers of dying cancer patients are affected by grief experiences and bereavement complications. Several approaches such as psycho-emotional care and an increase in spirituality have been suggested to diminish these complications. However, the knowledge about the effects of family-based dignity intervention and expressive writing on anticipatory grief in family caregivers of dying cancer patients is limited. This is a study protocol describing a hospital-based mixed-methods study on the effects of family-based dignity intervention and expressive writing on anticipatory grief in family caregivers of dying cancer patients.

**Methods:**

This mixed-methods study will be done in an embedded explanatory design with two quantitative and qualitative phases. In the first phase (quantitative), a randomized clinical trial will be done, in which 200 family caregivers of dying cancer patients will be randomly assigned to one of the four groups: family-based single dignity intervention (group 1), expressive writing intervention (group 2), combined family-based single dignity intervention and expressive writing (group 3), and control (group 4). At baseline, 1 week and 2 weeks after the interventions, anticipatory grief will be assessed by a 13-item anticipatory grief scale. After the quantitative phase, the qualitative phase will be conducted through the conventional content analysis approach of Granheim and Lundman, in which an individual semi-structured interview will be taken from participants in the first phase to collect data on their experiences on interventions. Finally, data from the quantitative and qualitative phases will be analyzed and discussed.

**Discussion:**

Family caregivers of dying cancer patients usually experience depression, anxiety, and psychological distress due to isolation and inadequate social support. Psychological interventions such as dignity and expressive writing interventions may help caregivers to obtain a better understanding of themselves and to increase their abilities to cope with caregiving difficulties. Therefore, there is a need for a comprehensive study confirming the effects of mentioned interventions on family caregivers of dying cancer patients.

**Trial registration:**

Iranian Registry of Clinical Trials (www.irct.ir) identifier: IRCT20210111050010N1. Date of trial registration: Feb 6, 2021. This is the first version of this protocol.

**Supplementary Information:**

The online version contains supplementary material available at 10.1186/s13063-021-05718-3.

## Introduction

The diagnosis of cancer is an unpleasant and unbelievable experience for every person, and it imposes a high burden on the patient and his/her families [1–3]. As cancer progresses, patients became physically and psychologically weaker, and their dependency on family increases [4]. In addition to the dependency, the caregiving responsibilities of patients are often transferred to family caregivers who are usually spouses, adult children, mother, or father [4, 5]. Therefore, family caregivers, particularly those who take care of dying cancer patients, usually are confronted with several social, physical, and psychological difficulties, which may adversely affect their health [6, 7]. It has been shown that more than 35% of family caregivers experience psychological disorders and feelings of helplessness, hopelessness, and anticipatory grief [8, 9]. This is the reason that caregivers are considered as “second-order patients,” who need supportive care [10].

The religious culture of Iranian caregivers makes a strong relationship between caregivers and patients [11]. For this reason, caregivers look at their responsivities as moral commitment and divine duty [12]. Nevertheless, this religious belief may cause them to hide their needs and caregiving problems [13]. Moreover, there are no specific social organizations in Iran to support caregivers and to diminish their problems [14]. Therefore, family caregivers in Iran, compared with those from developed countries, may be at a higher risk of psychological disorders or anticipatory grief.

Anticipatory grief is one of the important problems among family caregivers [6]. They usually think about the threat of death or separation that can initiate a grief reaction, while the patient is physically present and needed of care [15]. Anticipatory grief is a “safeguard against the impact of a sudden death notice” that helps caregivers to cope with bereavement [16]. However, anticipatory grief is a highly stressful and ambivalent experience that may be a reason for the incidence of psychological distress among caregivers [17, 18]. In addition, this feeling may be transferred to patients and affect their treatment process. Therefore, caregivers should try to control their feelings and refrain from expressing grief for cancer patients [19, 20].

Several psychological interventions have been proposed to support caregivers in their challenges [10, 21–23]. Recently, it has been shown that family-based dignity intervention and expressive writing are effective approaches in the treatment of psychological disorders [10, 24]. Family-based dignity intervention is a spiritual-psychological intervention taken from dignity therapy methods [25]. This supportive intervention helps caregivers to strengthen the sense of hope in themselves and give them an opportunity to talk about their successes, aspirations, and plans [26]. Expressive writing intervention includes sessions of solitary and unlimited writing about positive and negative feelings and experiences caused by stressful events [27, 28]. Overall, talking about successes, aspirations, plans, and also expressing feelings through writing is an appropriate strategy to enhance well-being and may promote the ability of caregivers to cope with chronic grief.

Limited data are available on the effects of family-based dignity intervention and expressive writing on anticipatory grief in family caregivers of dying cancer patients. In addition, most studies assessing the effects of mentioned interventions had been done with a quantitative or to a lesser extent with a qualitative research methodology that both contain several limitations and strengths [10, 29, 30]. Mixing the qualitative and quantitative methods can cover the limitations and bring together the strengths of both methods [31, 32]. Recently, mixed-methods methodology, defined as mixing both qualitative and quantitative methodologies, has been introduced and has captured the interest of healthcare researchers [33]. The mixed-methods approach in interventional studies helps researchers to assess the experiences of participants throughout the intervention period, and in this way, researchers can present a better conclusion than the quantitative-only and qualitative-only research approaches [33, 34]. Therefore, in the current mixed-methods study, we will first conduct a four-arm randomized controlled trial to assess the effects of (1) combined family-based dignity intervention and expressive writing, (2) family-based dignity intervention alone, (3) expressive writing alone, and (4) routine care on anticipatory grief of family caregivers of dying cancer patients and to compare the interventions to each other, and then, we will perform a qualitative study to assess the experiences of participants throughout the intervention period. In total, the overall aim of this study is which intervention is better for controlling anticipatory grief of family caregivers of cancer patients.

## Methods

### Participants

This mixed-methods study has an embedded explanatory design with one quantitative and one qualitative phase [35]. We will first conduct a four-arm randomized controlled trial to quantitatively evaluate the effects of (1) combined family-based dignity intervention and expressive writing, (2) family-based dignity intervention alone, (3) expressive writing alone, and (4) routine care on anticipatory grief of family caregivers of dying cancer patients. Then, we will assess the experiences of participants in the quantitative phase through qualitative interviews with them. In this single-center study, we will include family caregivers of dying cancer patients who refer to the oncology center of Firozgar hospital affiliated to the Iran University of Medical Sciences, Tehran, Iran, to receive medical and palliative care for their patients.

### Inclusion criteria

We will include caregivers with the following criteria: (1) caregivers who are the first-degree relatives [i.e., parents, offspring, and siblings] of cancer patients and have the most responsibility for caregiving of them during the last 3 months, (2) those who are caregiving of the cancer patients who are dying or critically ill according to the opinion of the treating physician, and (3) caregivers aged ≥18 years.

### Non-inclusion criteria

**Fig. 1:**
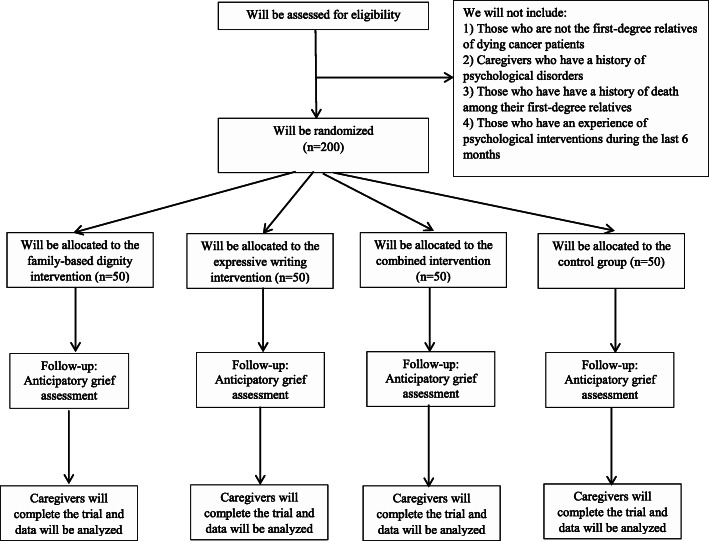
Study flowchart

We will not include caregivers who have a history of psychological disorders and those who have a history of death among their first-degree relatives. Also, caregivers who are not the first-degree relatives of dying cancer patients and those who have experience of psychological interventions (particularly dignity and expressive writing interventions) during the last 6 months will not be included. Also, we will exclude those caregivers who are not willing to continue each phase (quantitative and qualitative) throughout the study. Figure 1 shows the study flowchart.

### Ethics

All caregivers will read the terms written in informed consent, and optionally, they can accept to participate in the current study (Supplemental File 1). Principal investigators (MR and TNG) will be responsible for obtaining informed consent. The ethics committee of the Iran University of Medical Sciences approved the study (code: IR.IUMS.REC.1399.1097). Moreover, the quantitative phase of this study that is a randomized clinical trial was registered in the Iranian Registry of Clinical Trials (www.irct.ir) with code IRCT20210111050010N1.

### Study design for the quantitative phase

In the first phase of the current study (quantitative), a four-arm randomized controlled trial will be done. After recruiting family caregivers of dying cancer patients according to the inclusion criteria, they will be randomly assigned to one of the four groups: family-based single dignity intervention (group 1), expressive writing intervention (group 2), combined family-based single dignity intervention and expressive writing (group 3), and controls (group 4). Recruitment and random allocation will be done simultaneously. Allocation concealment will be done using the blocked randomization method. For this, we will use six blocks, each with a block size of 4 (A: group 1, B: group 2, C: group 3, and D: group 4), that the order of the letters in these blocks will be different (e.g., ABCD, ACDB). Then, a code ranging between 1 and 6 will be assigned to each of these six blocks. For allocating each four caregivers, at first, we will select one of the six blocks using random numbers obtained from an online randomizer (https://www.randomizer.org), and then, caregivers will be assigned to the four intervention groups according to the order of letters in the selected block. To ensure sufficient participant recruitment to reach the required sample size, recruitment and random allocation will continue until all groups become complete. Therefore, the enrollment period may extend over 12 months. It should be noted that random allocation will be done by a person who is unaware of the aim of our study.

After the random allocation, sessions related to the interventions (family-based single dignity intervention and expressive writing) will take place at the time that will be set with caregivers. To avoid sharing experiences between caregivers, we will try to provide a situation that caregivers do not have contact with each other. For this, we will include a maximum of four caregivers in a day from different rooms of the oncology center in Firozgar hospital (study setting) and we will set a specific time for each caregiver so that there is no possibility of contact and sharing experiences with each other. Also, after discharging the four previously included caregivers from the hospital, we will include the other new four caregivers to minimize their contact. On the other hand, recruitment will be stopped until discharging the four previously included caregivers. Caregivers in all intervention groups will receive routine care such as family counseling and meaning therapy, and they will be asked not to participate in other psychotherapy programs. Since the interventions used in the current study are interview-based, we cannot blind the investigators and caregivers to the interventions.

**Fig. 2:**
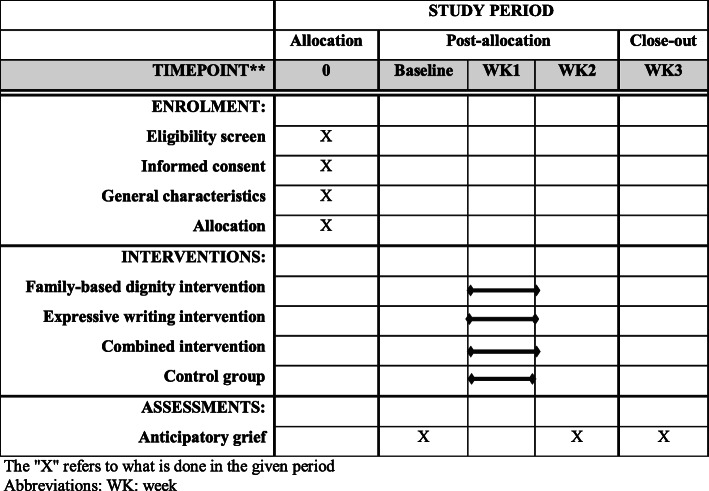
Standard Protocol Items: Recommendations for Interventional Trials (SPIRIT) chart of the enrolments and assessments during randomized controlled trials. The “*X*” refers to what is done in the given period. Abbreviations: WK week

Before the intervention session and 1 week and 2 weeks after the interventions, anticipatory grief will be assessed by a 13-item anticipatory grief scale (AGS-13). The mean of AGE-13 score at baseline and throughout the study will be considered as the main outcome variable. The diagram for Standard Protocol Items: Recommendations for Interventional Trials (SPIRIT) is shown in Fig. 2.

### Sample size calculation

Considering the type I error of 5% (*α*= 0.05) and type II error of 20% (*β*= 0.20, power = 80%) and anticipatory grief score as the key variable, we manually, without the use of any software, calculated the required sample size using the following formula [36, 37]:

$$ n=\frac{2\left[{\left(\mathrm{a}+\mathrm{b}\right)}^2\times {\upsigma}^2\right]}{{\left({\mu}_1-{\mu}_2\right)}^2} $$

*n* = sample size in each group

*μ*_1_ = mean for anticipatory grief score in group 1 (considered as 78.17 based on the study of Garand et al. [38])

*μ*_2_ = mean for anticipatory grief score in group 2 (considered as 64.49 based on the study of Garand et al. [38])

*σ* = variance (SD) for the mean of anticipatory grief score, which was considered as 23.1 (the average SDs reported for anticipatory grief score in the 2 groups of Garand et al. study [38]).

*a* = conventional multiplier for alpha = 0.05 that was 1.96

*b* = conventional multiplier for power = 0.80 that was 0.842

Overall, based on the formula and given a 10% drop-out in each group, we will need a sample size of 50 caregivers for each intervention group.

### Family-based single dignity intervention

This single intervention will be delivered by a researcher who is experienced in counseling. This intervention will be done based on the protocol introduced by Ho et al., through which, a 60–90-min intervention interview session will take place for each caregiver in a private room in the palliative care center [29]. In this session, caregivers will be asked to answer 12 open-ended questions related to the Ho et al. protocol. The questions focus on eliciting caregivers’ experiences of living with a cancer patient before and after the diagnosis of cancer. Also, these questions will help caregivers to review the beautiful memories of living with a cancer patient and express their hopes, wishes, and desired expectations. The responses to these questions will be audio-recorded and then will be transcribed verbatim in a manuscript for each caregiver. The manuscript will be checked with caregivers and, if needed, corrections will be done. A copy of the manuscript will be delivered to caregivers.

### Expressive writing intervention

This intervention will be done based on the Pennebaker method [39]. Before the intervention, caregivers will be instructed by a trained researcher in order to do this writing. The researcher will receive training on expressive writing by participating in a writing art workshop. Caregivers in this group will be asked to write about a prompt, and they will be instructed to “really let go and explore their very deepest emotions and thoughts.” They will be also told to write about either negative or positive family memories and describe their experiences of caregiving in the present and past time. This writing should be done three times (in a week) lasting 20 min. In that week, the researcher will remind the writing process using a phone call. They are also assured that they do not need to worry about sentence structure or grammar when writing. After 1 week of opportunity for expressive writing, the manuscript of caregivers will be received.

### Combined family-based dignity intervention and expressive writing

For this combined intervention, at first, the session related to family-based dignity intervention will be held and after that, training on expressive writing will be delivered to caregivers. After 1 week from the last session, the manuscript of caregivers will be received.

### Control group

Caregivers in the control group, as well as those in the intervention groups, will receive routine care such as family counseling and meaning therapy. In brief, meaning therapy is an integrative and positive existential approach for counseling and psychotherapy. Details on this therapeutic method are published elsewhere [40].

### Post-trial care

After finishing this study, we will recommend caregivers to receive routine care such as family counseling and meaningful therapy for having good mental health. Also, at the end of the study, we will assess whether combined interventions of family-based dignity and expressive writing or each intervention alone reduce anticipatory grief by 10 points, based on the AGS-13, compared to the routine care as a control intervention. The 10-point decrease or increase is considered a clinically and statistically significant change in anticipatory grief based on the study of Garand et al. [38] and also given the total AGS-13 scores ranging between 13 and 65.

### Anticipatory grief scale

The original form of anticipatory grief scale was designed by Theut et al. to assess anticipatory grief in spouses of Alzheimer’s patients [41]. This form consisted of 27 items measuring anticipatory grief on a Likert scale, ranging from 1 (strongly disagree) to 5 (strongly agree). The items should be summed into a total score ranging from 27 to 135 with a higher score indicating higher levels of anticipatory grief. However, 8 items (2, 5, 8, 11, 19, 22, 26, and 27) have a positive bearing and therefore must be reversed before the total score is calculated. In addition to the total score of anticipatory grief, the score of seven subscales including anger, guilt, anxiety, irritability, sadness, feelings of loss, and decreased ability to function in performing usual tasks can be reached from this scale. In 2019, Holm et al. [42] introduced a short form of AGS containing 13 items and was named AGS-13 (items 1, 3, 4, 7, 10, 15, 16, 18, 20, 21, 23, 24, and 25 of the original form). This scale specifically measures a total score of anticipatory grief in family caregivers of cancer patients and also consists of two subscales, named “Behavioral reactions” and “Emotional reactions,” which capture the behavioral and emotional reactions of grief in caregivers participating in palliative care. To obtain a total score of anticipatory grief in AGS-13, like the original form, the items should be summed into a total score ranging between 13 and 65. Higher scores indicate higher anticipatory grief in caregivers of cancer patients. No item should be reversed before calculating the total score. To increase the data quality obtained from the AGS-13, investigators will give a brief explanation about the questionnaire and its items to participants. Also, we have no concern about the effect of baseline AGE-13 responses on the second and third assessments (1 week and 2 weeks after the interventions) because Holm et al. reported that the questionnaire has sufficient sensitivity that means any true changes in anticipatory grief result in a change in the total score of AGS-13 [42].

### Translation, validity, and reliability of AGS-13

#### Translation

Since the AGS-13 was not used in Iran, we will translate it to Persian based on the method proposed by Guillemin [43]. At first, the permission to use and translate the AGS-13 will be gathered from its developer (Holm et al.) [42]. Then, the English version of the instrument will be translated to Persian by two independent health professionals fluent in English and Persian languages and familiar with the concepts of the questionnaire. After that, an expert panel will assess the translations and select the best translation of each item, and finally, a single form will be produced. Then, the initial Persian form of the tool will be translated back to English by two other qualified persons and the best backward translations will be chosen by the same expert panel afterward. The final backward translation form will be sent to the developer of the tool, to be reviewed for probable inadequacy of words and concepts. Consequently, the confirmed Persian version of the tool will be used for the current study.

#### Validity

Content validity (CV) will be used to assess the validity of AGS-13. The assessment of CV will be done using two qualitative and quantitative methods. In the qualitative method, the opinion of 11 experts in the fields of instrumentation and psychometric measurement, community health nursing, and specialist physicians in the field of psychology and oncology will be received in order to observe and assess grammar, wording, item allocation, and scaling. Then, the quantitative CV will be examined using the Content Validity Ratio (CVR) and Content Validity Index (CVI) [44, 45].

#### Internal consistency

Cronbach’s alpha coefficient will be used to measure the internal consistency of the AGS-13 using SPSS software (version 18).

#### Test-retest reliability

To measure the stability of the AGS-13, 30 caregivers will be asked to fill the scale two times with a 2-week interval. These caregivers will not participate in the main study. Test-retest reliability will be tested using the intra-class correlation coefficient (ICC) with a 95% confidence interval (CI). *P* values less than 0.05 will be considered significant.

#### Other information

Demographic data including age, sex, marital status, education, medical history, and medication will be collected at the baseline.

### Statistical analysis for the quantitative phase

All collected data will be coded and then will be entered into the SPSS software version 18 (SPSS, Inc., Chicago, IL, USA) for performing statistical analysis. To ensure the accuracy of the dataset, it will be double-checked by another investigator. The analyses will be performed on the basis of an intention-to-treat (ITT) approach. Therefore, missing values will be treated according to the last-observation-carried-forward method. The Kolmogorov-Smirnov test will be used to examine the normal distribution of quantitative variables. We will normalize the non-normally distributed variables using log transformation. To detect differences in quantitative and categorical variables between the intervention groups, we will use the analysis of variance (ANOVA) and chi-square test, respectively. To determine the effect of family-based dignity intervention and expressive writing on anticipatory grief, we will use repeated measures ANOVA. In this analysis, the intervention groups (family-based dignity intervention, expressive writing, combined intervention, and control groups) will be considered as the between-subjects factor and the time points (before and 1 week and 2 weeks after the interventions) will be considered as the within-subjects factor. Also, to assess differences between the intervention groups in terms of mean changes of anticipatory grief, the multivariate analysis of covariance (ANCOVA) will be applied by considering baseline measurements and variables different between the groups as covariates. *P* value<0.05 will be considered as a significant level.

### Study design for the qualitative phase

The first aim of this phase is to evaluate the experiences of participants during the family-based dignity intervention and expressive writing and what they have felt on the days after the interventions. Second, we will assess the extent of the effects of interventions on caregivers and whether the possible changes in the total score of anticipatory grief are due to the interventions or other factors are involved. Finally, we will aim to investigate whether a significant reduction in anticipatory grief of caregivers results in better care for cancer patients. The qualitative phase will be done after the completion of the first phase and based on the conventional content analysis approach suggested by Lundman and Granheim [46]. Some caregivers participating in the first phase will be selected for the qualitative phase according to the purposeful sampling that is widely used in qualitative studies [47]. There are several different purposeful sampling strategies; however, in the current study, we will use criterion sampling that appears to be used most commonly in qualitative research [47]. In this sampling method, we will identify the information-rich caregivers related to the patient care based on the pre-defined criteria: (1) those caregivers that have a 10-unit change in anticipatory grief at the end of the quantitative phase compared to baseline and (2) caregivers with the score unchanged throughout the first phase. Also, we will include those caregivers that have enough time and are willing to contribute in the qualitative phase.

Qualitative data will be collected using individual, face-to-face, in-depth and semi-structured interviews, containing open questions, by the main researcher. The desired questions used in the interview will be designed based on the findings obtained from the quantitative phase. We will set a time for the interview with each participant according to his/her preferences. Before the interview, the aims of the research will be expressed to the caregivers and they will be assured that the data collected are anonymous and confidential and are used only for this study. All interviews will be carried out in a suitable environment based on the participants’ preferences and without someone other than the interviewees. Because of the coronavirus epidemic, interviews may be conducted online using Skype or WhatsApp applications. In each interview session, we will ask some open questions from participants given their intervention types. The interview will be started with open-ended questions: “How were the face-to-face meetings we had together?”, “Please describe your experiences in relation to the intervention”, “Compared to before the intervention, have you had any changes in your relationships with your patient?”, and “Please talk about what happened to you throughout the study”. Following questions (e.g., can you give an example?/please explain more) will be also used based on the responses of participants to lead the interview to clarify caregivers’ perceptions. Also, if the question is not clear for participants, the researcher will use some examples to clarify the question. Interview data will be entirely recorded in three ways: voice recording and taking notes during and after the interview. Of note, interviews will be recorded with the consent of the caregivers. Interviews will continue until the data saturation is reached.

### Data analysis for the qualitative phase

We will analyze the qualitative data using qualitative content analysis according to the Graneheim and Lundman method [46]. Accordingly, the data will be analyzed immediately after each interview. The audio files will be heard several times, and then, they will be converted into a writing format. The notes were studied line-by-line to extract the codes. At first, target words that contain the main concepts will be determined. The researcher continued reviewing the text by taking notes from the initial assessment until the major codes will be elicited. After that, the code labels indicating >1 key thought will be directly extracted and determined. Finally, the extracted codes will be categorized into themes and main categories according to their differences and relationships. We will also determine the subcategories of the codes based on differences and similarities.

## Discussion

Family caregivers of dying cancer patients usually experience different psychological disorders including depression, anxiety, and psychological distress due to isolation and inadequate social support [6, 7, 48]. Anticipatory grief is another complication of caregiving terminally ill patients [49]. Caregivers usually think about how much separation and loneliness can be hard and this thought can initiate a grief reaction, while the patient is physically present and in need of care [15]. Therefore, if these disorders are not managed, they can adversely affect the quality of caregiving and also caregivers’ health [50].

Different strategies such as pharmacological treatments and psychological interventions have been designed to control psychological disorders [51, 52]. Compared to pharmacological treatments, psychological interventions including psychological counseling have fewer complications and can help in the complete treatment of the disorders [51]. Several psychological interventions have been suggested to help caregivers to control their difficulties [10, 21–23]. However, little attention has been paid to family-based dignity intervention and expressive writing [10, 24]. These supportive interventions may help caregivers to strengthen the sense of well-being in themselves and give them an opportunity to talk or write about their successes, aspirations, and plans [26–28]. These interventions have no complications and are much more cost-effective compared to medications.

### Strengths

This is the first mixed-methods study investigating the effects of family-based dignity intervention and expressive writing on anticipatory grief in family caregivers of dying cancer patients through mixing quantitative and qualitative assessments. Also, it should be noted that the interventions are of low cost and can easily be carried out in palliative care centers. In this study, caregivers are assigned to four groups using block randomization. The sample size of this study is adequate and provides sufficient power to detect the efficacy of interventions. In addition to the effects of family-based dignity intervention and expressive writing alone, the combined effects of these interventions are examined in a separate intervention group.

### Limitations

Despite using validated questionnaires for the assessment of anticipatory grief, misclassification of caregivers in terms of this variable cannot be fully excluded. Since the interventions used in the current study are interview-based, we cannot blind the caregivers to the interventions. Although we try to control for variables different between the groups in statistical analysis, we cannot entirely exclude the effects of residual confounders or differences on our findings.

## Supplementary Information

**Additional file1: Supplemental file 1.** Consent form

## Data Availability

Only investigators will have access to the final trial dataset.

## Abbreviations

AGS: Anticipatory Grief Scale
