# Supplementary material for: Effects of family-based dignity intervention and expressive writing on anticipatory grief of family caregivers of patients with cancer: a study protocol for a four-arm randomized controlled trial and a qualitative process evaluation
Source: Trials. 2021 Oct 28;22:751. doi: 10.1186/s13063-021-05718-3 (PMC8552199; doi:10.1186/s13063-021-05718-3)
Supplement: Supplementary file 1 — Additional file1: Supplemental file 1. Consent form [file 13063_2021_5718_MOESM1_ESM.docx]

**Supplementary File 1: Consent Form**

عنوان طرح : بررسی تاثیر مداخله کرامت خانواده محور و بیان نوشتاری بر اندوه مراقبین خانوادگی افراد با سرطان: یک مطالعه ترکیبی لانه گزین شده

بدین وسیله از شما جهت شرکت در پژوهش فوق الذکر دعوت به عمل می آید. اطلاعات مربوط به این پژوهش در این برگه خدمتتان ارائه شده است و شما برای شرکت یا عدم شرکت در این پژوهش آزاد هستید.

شما مجبور به تصمیم گیری فوری نیستید و برای تصمیم گیری در این باره می توانید سوالات خود را از تیم پژوهشی بپرسید و با هر فردی که مایل باشید مشورت نمایید. قبل از امضای این رضایت نامه مطمئن شوید که متوجه تمامی اطلاعات این فرم شده اید و به تمام سوالات شما پاسخ داده است.

مجری پژوهش:

دکتر سیده نعیمه سیدفاطمی

**1.من می دانم که اهداف این پژوهش عبارتند از:**

بررسی تاثیر مداخله کرامت خانواده‌محور و بیان نوشتاری بر اندوه مراقبین خانوادگی مددجویان با سرطان خواهد بود.

من میدانم هدف پژوهش انجام مداخلاتی خواهد بود و به بهبود مشکلات مراقبتی و غم و اندوه اینجانب کمک خواهد کرد. من می دانم اگر مداخلات حمایتی بر مشکلات مراقبتی موثر باشد، آیندگانی که دارای مشکلات مشابه با من هستند می توانند از نتایج پژوهش بهره مند شوند.

**2.من می دانم شرکت من در این پژوهش** کاملا داوطلبانه است و مجبور به شرکت در این پژوهش نیستم به من اطمینان داده شد که اگر حاضر به شرکت در این پژوهش نباشم، از مراقبت های معمول تشخیصی و درمانی محروم نخواهم شد و رابطه درمانی من بامرکز درمانی و پرشک معالج دچار اشکال نشود.

**3.من می دانم که** حتی پس از موافقت با شرکت در پژوهش می توانم هر وقت که بخواهم، پس از اطلاع به مجری، از پژوهش خارج شوم و خروج من از پژوهش باعث محرومیت از دریافت خدمات درمانی معمول برای من نخواهد شد.

**4.نحوه ی همکاری اینجانب در این پژوهش به این صورت است:**

نحوه همکاری ام کاملا داوطلبانه است. اطلاعاتی در مورد مداخله مورد نظر به من داده خواهد شد و در طی یک یا دو جلسه نشست (حداکثر 90دقیقه ) سوالاتی از من پرسیده خواهد شد و مجددا جهت تکمیل پرسشنامه به صورت حضوری و مجازی با تیم تحقیق ارتباط خواهم داشت. زمان و مکان نشست را خودم تعیین خواهم کرد و هزینه رفت و امد به محل پژوهش بر عهده تیم تحقیق خواهد بود.

**5.منافع احتمالی شرکت اینجانب در این مطالعه به شرح زیر است:**

اطلاع از نتایج مطالعه و کمک به مشکلات مراقبتی مراقبین خانواگی، کاهش حس اندوه و سازگاری با فقدان احتمالی و همچنین در اختیار گذاشتن تجربیات مراقبتی اینجانب به اعضای سیستم مراقبتی بهداشتی جهت پی ریزی حمایت های همه جانبه از بیماران و خانواده آنها کمک خواهد کرد

**6.آسیب ها و عوارض احتمالی شرکت در این مطالعه به این شرح است:**

هیچگونه آسیبی متحمل نخواهم شد.

**7.در صورت عدم تمایل به شرکت در مطالعه روش معمول درمانی برای من ارائه خواهد شد که منافع و عوارض آن به این شرح است:**

در این صورت روند معمول کارم در مرکز ادامه دارد و وقفه ای در مراقبت از عضو خانواده ام به وجود نخواهد آمد.

**8.من می دانم که** دست اندرکاران این پژوهش، کلیه اطلاعات مربوط به من را نزد خود به صورت محرمانه نگه داشته و فقط اجازه دارند نتایج کلی و گروهی این پژوهش را بدون ذکر نام و مشخصات اینجانب منتشر کنند.

**9.می دانم که** کمیته اخلاق در پژوهش با هدف نظارت بر رعایت حقوق اینجانب می تواند به اطلاعات من دسترسی داشته باشد.

**10.من می دانم که** هیچ یک از هزینه های انجام مداخلات پژوهشی به شرح ذیل بر عهده من نخواهد بود.

هزینه های ایاب و ذهاب بر عهده مراقب نخواهد بود و هیچ هزینه ای را متحمل نخواهم شد.

**11. مشخصات فردی که جهت پاسخگویی به اینجانب معرفی شد** و به من گفته شد تا هر وقت مشکلی یا سوالی در رابطه با شرکت در پژوهش مذکور پیش آمد با ایشان در میان بگذارم و راهنمایی بخواهم. نام و آدرس و شماره تلفن ثابت و همراه ایشان به شرح زیر به من ارائه شد.:

نام و نام خانوادگی سیده نعیمه سیدفاطمی

آدرس: دانشکده پرستاری و مامایی دانشگاه علوم پزشکی ایران

تلفن ثابت: 43651722

**12.من می دانم که** اگر در حین و بعد از انجام پژوهش هر مشکلی اعم از جسمی و روحی به علت شرکت در این پژوهش برای من پیش آمد درمان عوارض آن و غرامت مربوطه بر عهده مجری خواهد بود.

**13.من می دانم اگر** اشکال یا اعتراضی نسبت به دست اندرکاران یا روند پژوهش دارم میتوانم با کمیته اخلاق در پژوهش دانشگاه علوم پزشکی ایران به آدرس : تهران،دانشگاه علوم پزشکی ایران ، بزرگراه شهید همت غرب بین تقاطع شیخ فضل الله و شهید چمران ستاد مرکزی طبقه 5 معاونت تحقیقات و فناوری با شماره 86702530 تماس گرفته و مشکل خود را به صورت شفاهی یا کتبی مطرح نماییم.

**14.این فرم اطلاعات و رضایت آگاهانه در دو نسخه تهیه شده** و پس از امضا یک نسخه در اختیار من و نسخه دیگر در اختیار مجری قرار خواهد گرفت.

- اینجانب موارد فوق الذکر را خواندم و فهمیدم و بر اساس آن رضایت آگاهانه خود را برای شرکت در این پژوهش اعلام میکنم

- اینجانب سیده نعیمه سیدفاطمی خود را ملزم به اجرای تعهدات مربوط به مجری در مفاد فوق دانسته و متعهد میگردم در تامین حقوق و ایمنی شرکت کننده در این پژوهش تلاش نمایم.

مهر و امضای مجری پژوهش امضای شرکت کننده

دکتر سیده نعیمه سیدفاطمی

دکتر طاهره نجفی

مسعود رضائی
